# Physical and Emotional Benefits of Different Exercise Environments Designed for Treadmill Running

**DOI:** 10.3390/ijerph14070752

**Published:** 2017-07-11

**Authors:** Hsiao-Pu Yeh, Joseph A. Stone, Sarah M. Churchill, Eric Brymer, Keith Davids

**Affiliations:** 1 Centre for Sports Engineering Research, Sheffield Hallam University, Sheffield S10 2BP, UK; hwbkd@exchange.shu.ac.uk; 2 Academy of Sport and Physical Activity, Sheffield Hallam University, Sheffield S10 2BP, UK; hwbjs9@exchange.shu.ac.uk (J.A.S.); hwbsc3@exchange.shu.ac.uk (S.M.C.); 3 Institute of Sport, Physical Activity and Leisure, Leeds Beckett University, Leeds LS1 3HE, UK; E.Brymer@leedsbeckett.ac.uk

**Keywords:** green physical activity, environmental design, happiness, ecological dynamics, indoor exercise environments

## Abstract

(1) Background: Green physical activity promotes physical health and mental wellbeing and interesting questions concern effects of this information on designing indoor exercise environments. This study examined the physical and emotional effects of different nature-based environments designed for indoor treadmill running; (2) Methods: In a counterbalanced experimental design, 30 participants performed three, twenty-minute treadmill runs at a self-selected pace while viewing either a static nature image, a dynamic nature image or self-selected entertainment. Distance ran, heart rate (HR) and five pre-and post-exercise emotional states were measured; (3) Results: Participants ran farther, and with higher HRs, with self-selected entertainment compared to the two nature-based environment designs. Participants attained lowered anger, dejection, anxiety and increased excitement post exercise in all of the designed environments. Happiness increased during the two nature-based environment designs compared with self-selected entertainment; (4) Conclusions: Self-selected entertainment encouraged greater physical performances whereas running in nature-based exercise environments elicited greater happiness immediately after running.

## 1. Introduction

Physical inactivity has been identified as the fourth leading risk factor for global mortality, associated with approximately 3.2 million deaths each year and implicated in the prevalence of non-communicable diseases such as cancer and cardiovascular issues [1]. As the proportion of the world’s population living in urban environments is increasing, this has become an important group to target with strategies for increasing physical activity (PA) uptake, effectiveness and adherence [2,3]. Moreover, the increasing trend for exercising requires better understanding, how considering best to design these settings to maximise benefits. This paper examines the physical and emotional effects of exercising in different indoor PA environments. 

Urban environments, high-density traffic, low air quality, a lack of parks, sports/recreation facilities and fear of crime in outdoor areas [4] might inhibit urban dwellers from exercising outside. Bad weather, lack of time or shorter daytime light can also act as barriers discouraging people from exercising outside, especially during winter. As a result, gyms, homes or private exercise centres can become preferred venues for PA because of the reduced concerns about safety and increased availability. In fact, the exercise gym is the most preferred PA environment [5]. Depending on the exercise activity and available facilities, exercisers physically and psychologically engage with their activity in different ways. For example, exercisers might prefer to exercise while watching television programmes, news or movies, or listening to music available in the external environment or on their own music devices. These varying media types offer different information sources which in turn influences exercisers’ perceptions, performance and experiences. For example, listening to music during treadmill running can influence runners’ performance compared to watching television programmes [6]. Different forms of the same entertainment might also result in different PA outcomes. For example, fast or loud music can encourage treadmill runners to run faster or for a longer time [7,8,9], whereas slow or quiet music has not been associated with any beneficial physical outputs for treadmill runners [9]. Hence, identifying the information in PA contexts that more effectively engages exercisers and maximises physical and mental exercise benefits is important for ensuring the effective design of PA environments. 

Nature has been promoted as integral to human health and wellbeing, being associated with the capacity to augment physical, cognitive and emotional wellbeing. For example, people who live in greener environments report better perceived health [10] and viewing awesome nature scenes has been associated with mood improvement [11]. When coupled with PA, the context of nature has been associated with lowered blood pressure [12,13], reduced perceived exertion [14,15], enhanced self-esteem [13,16], mood [13,14,16] and enjoyment [17,18,19] as well as reduced anxiety [20,21,22,23] and stress [23,24,25]. Furthermore, viewing static and dynamic images of nature while participating in indoor physical activities, such as running on a treadmill or cycling on an exercise bike (defined as ‘in the presence of nature’) has also demonstrated physical and mental benefits, such as lowered blood pressure [12,13], lowered perceived exertion [14,15], improved direct attention [26], mood [13,14,15], self-esteem [13,27], affective valence and exercise enjoyment [19].

A meta-analysis undertaken by Bowler and colleagues indicated that the most commonly reported benefit of PA in the presence of nature (indoors or outdoors) was the enhancement of emotions [28]. A number of theoretical perspectives have been proposed as useful for understanding how this might come about. Attention Restoration Theory suggests that nature environments have a restorative effect on the brain's ability to focus. Whereas, the Stress Recovery Theory [29] posits a healing power of nature that lies in an unconscious, autonomic response to natural elements that can occur without recognition and most noticeably in individuals who have been stressed before the experience [30]. Further, The Biophilia hypothesis assumes that humans have affiliations to nature [31]. Despite these explanations, theoretical perspectives have largely ignored the role of nature in PA design. Interpretations have been limited to psychological and cognitive responses, which provide a narrow perspective on the beneficial effects of nature. However, the individual, the type of PA and the environment in which the PA is performed all play influential roles in the emergence of behaviour [2]. 

Ecological dynamics has been proposed as an effective framework for understanding the relationship of individuals with the environment during PA [2,6]. Ecological dynamics emphasises that the realisation of affordances underpins observed effects of PA [3,6]. The notion of affordances highlights that the relationship between a perceiver’s capabilities and an environment supports opportunities (both good and bad) that facilitate a given activity [32]. To perceive an affordance is to detect an environmental property that provides an opportunity for action and it is specified in the surrounding environment available to perceivers [32]. Therefore, when performing an activity, an individual is constantly and actively detecting various types of information, such as olfactory, acoustic, haptic and visual from the environment and utilising information that is most functional during interactions. For example, when you run along a river, you might notice fish swimming in the river, but it is not functionally relevant to your running. However, a puddle on the pathway or a slippery surface near the water’s edge might be very relevant for the way that your emotions and physical actions emerge. In this way, the perception-action relationship is a reciprocal and continuous cycle that underpins human behaviour. Based on the concept of affordances, people exercising in the same physical environment, might detect or utilise different information sources which would accrue various effects on their behaviours, according to individual differences. A static scene is a frozen moment and may contain limited information for participants to utilise compared to a dynamic display which offers continuous and richer information for affordances. Hence, the functionality offered by these two types of information may differ, although such variations might not have linear effects. Previous work has examined these two types of displays and found a non-parallel relationship between static and dynamic displays on preference rating, epistemic and evaluative variables with no PA involvement [33]. When applied in a PA environment, the effects of viewing static and dynamic displays during PA remain unclear. 

To examine this key idea, we provided three conditions which afforded (offered) different information sources for exercisers performing PA in the same physical setting, through different environmental designs. We examined emotional and physical outcomes related to exercising when viewing two types of nature-based designs and when participants were able to choose their habitual, preferred entertainment. The two nature conditions were designed with visual-only information, whereas in the self-selected entertainment condition, participants were able to choose visual, acoustic or visual-acoustic information. In all three PA designs, participants were instructed to run at their own comfortable pace and they were allowed to change their running speed at any time during the activity. Therefore, the imposed speed of the run, e.g., the intensity of the run, was not allowed to contaminate the findings. Allowing participants to self-adjust running intensity during an experimental condition is more representative of their typical experiences during PA. It is, therefore, more likely to enhance knowledge about how to design a more appealing indoor exercising (e.g., treadmill running) environment with typical affordances of different activity contexts. We sought to investigate these PA designs to understand whether any were more likely to be beneficial for constraining experiences of physical health and mental wellbeing [34,35]. Therefore, the aim of this paper was to examine physical and emotional effects of PA in different exercise environments with and without nature-based affordances without controlling the intensity of PA. We hypothesised that participants would accrue more emotional benefits when viewing dynamic image than a static image, due to the dynamic qualities of the information present in that type of display, however, it was expected that self-selected entertainment would result in better physical performance, based on previous research findings. 

## 2. Materials and Methods 

### 2.1. Participants

Thirty participants (mean ± SD: 18 males and 12 females; age 27.5 ± 9 years; mass 67.6 ± 11.1 kg; stature 173.7 ± 8.2 cm; BMI 22.2 ± 2.1) were recruited. All participants gave their informed consent for inclusion before they participated in the study. The study was conducted in accordance with the Declaration of Helsinki, and the protocol was approved by the Ethics Committee of Sheffield Hallam University Research Ethics Committee of HWB-S&E-35. Twenty-four participants performed regular exercise completing more than 150 min a week, such as attending gym sessions, weightlifting, running, cycling and climbing. Six participants performed exercise irregularly or light exercise, such as power walking.

### 2.2. Study Design

**Figure 1:**
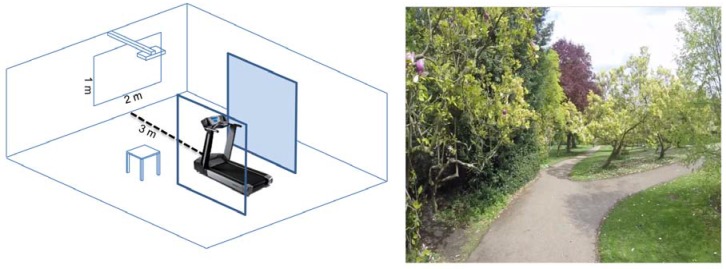
**Left:** The experimental setting, the treadmill is 3 m from the wall with projecting media and two partitions stand next to the treadmill [6]. The projected screen is 2 × 1 m; **Right:** The one single frame from the dynamic image condition used throughout the whole twenty-minute.

Two nature-based conditions, involving visual-only information of nature, were designed. The first involved a static image of nature and the second included a dynamic image. The dynamic image condition was a 20-minute digital video recording made at the Sheffield Botanical Gardens. The video recording was created by fixing a GoPro camera (Hero3+, GoPro, San Mateo, CA, USA) on the helmet of a person cycling along a series of paths within the gardens, capturing the trail through lawns, trees and flower beds on a sunny afternoon in spring. The video aimed to represent a first person perspective of moving through the gardens and it was filmed at 2.32 m/s to present a moderate exercise level [36]. The static image condition was composed of a single frame of the dynamic image to avoid discrepancies between images and was used throughout the twenty minutes physical activity period (see Figure 1). The third design, representative of popular gym conditions, consisted of self-selected, preferred entertainment where participants were able to choose preferences that included visual and/or auditory information. To focus on personal preferences of participants, there were no specific limitations imposed on the self-selected entertainment used for Gym exercise. Participants chose various entertainments, for example, listening to music (*N* = 23), watching television (e.g., BBC news/talk shows) or movies (e.g., Simpsons) with sound (*N* = 6) and viewing a picture (one person chose to view an image of friends). Television, movies and the static image of nature were presented on a wall-mounted monitor with a 2 × 1 m screen, situated 3 m in front of the treadmill (Figure 1). Music was presented either from wall mounted speakers or through participants’ own headphones. In each trial, participants performed a self-organised warm up for 5 min. The information panel on the treadmill was covered to ensure that participants were not able to view the distance of their run. The researcher was able to record these data by using the treadmill application program in a remote computer. There were two partitions on each side of the treadmill in order to control potential distractions by limiting participants' visible area to the forward plane.

### 2.3. Procedure

In a counterbalanced design, all participants were asked to perform a twenty-minute treadmill run at a comfortable self-selected speed in each design at a similar time of day (within a 4 h window). There was at least a seven-day gap between conditions to ‘wash out’ condition effects and avoid fatigue for each participant [26]. Participants were informed that they could change their speed at any time during the run. The information displayed on the control panel of the treadmill was covered, but participants could still change their speed by pressing a button on the treadmill control panel. Before the first trial, data on age, mass, stature and resting heart rate (HR) were collected. The distance run by participants in each 20-min session was recorded and HR data were recorded continuously (per second) for twenty minutes with a Polar HR watch (Polar RS400, Polar Electro, Kempele, Finland). The speed of the twenty-minute run was recorded by the researcher minute-by-minute (4 participants were excluded because of an incomplete data set). The Sport Emotion Questionnaire (SEQ) was used to examine people’s emotional states five minutes before the run and immediately after the run in each trial. The SEQ is a valid and reliable measure of sport-specific emotions [37], and has been effectively used in different exercise groups [38]. The SEQ is a 22-item measure for happiness, anxiety, dejection, anger and excitement. The Happiness subscale encompasses a person’s self-appraisal with regards to their progress towards a goal. It consists of four items, i.e., Pleased, Joyful, Happy and Cheerful. Anxiety is considered to reflect uncertainty regarding goal attainment and coping, and consisted of five items, i.e., Uneasy, Tense, Nervous, Apprehensive and Anxious. Dejection is a negative emotion characterized by feelings of deficiency and sadness and assessed by five items, i.e., Upset, Sad, Unhappy, Disappointed and Dejected. Anger can be channeled internally to self-blame and associated with feelings of depressions or externally toward the source of the frustration. This subscale consisted of four items: Irritated, Furious, Annoyed and Angry. Excitement is proposed to occur when a person has a positive expectation of his or her ability to cope and achieve goals in a challenging situation. Exhilarated, Excited, Enthusiastic and Energetic are the four items for measuring excitement in the SEQ. The SEQ is rated with a 5-point Likert scale, i.e., not at all (0), a little (1), moderately (2), quite a bit (3) and extremely (4). Scores for each subscale are determined by calculating the mean of its assessed items. 

### 2.4. Data Analysis

Data were analysed in SPSS version 22 (IBM, Chicago, IL, USA) and an alpha level of 0.05 was used to indicate significant difference levels, with Partial eta squared used for effect size calculations. Least Significant Difference (LSD) was used for post hoc analysis. The HR data were exported from the commercial software (Polar Pro trainer 5, Polar Electro, Kempele, Finland) and mean HR for each participant for the twenty minutes of the run was used for analysis. The mean HR value for every minute of the run for all participants in the three separate conditions was calculated. Six participants were removed from the HR analysis because of technical problems. Examination of the Shapiro-Wilk test revealed distance and HR were not normally distributed. Hence, two Friedman tests were used to statistically analyse the differences in the values of the distances run and HR. Scores of the five subscales of SEQ were calculated. Five, separate, two-way repeated measures analysis of variance (ANOVAs) (time × condition) were used to examine any differences on five subscales of the sport emotion questionnaire.

## 3. Results

### 3.1. Running Distance, Heart Rate and Speed

Descriptive data for running distances and heart rate (HR) for each condition are displayed in Table 1. Distance run was influenced by the designs, *F* (29) = 10.572, *p* < 0.05, *pƞ*^2^ = 0.2 with participants in the self-selected entertainment condition (3066.8 ± 688.5 m) running longer distances than the static image condition (2767.2 ± 662.6 m) (*p* < 0.05). HR was also affected by the three designs, *χ*^2^ (2) = 10.750, *p* < 0.05. Participants exercising with self-selected entertainment (Mdn = 149.11 bmp) achieved a higher HR than in the dynamic image condition (Mdn = 140.52 bmp, *p* < 0.05), and in the static image condition (Mdn = 142.03 bmp, *p* < 0.05).

**Figure 2:**
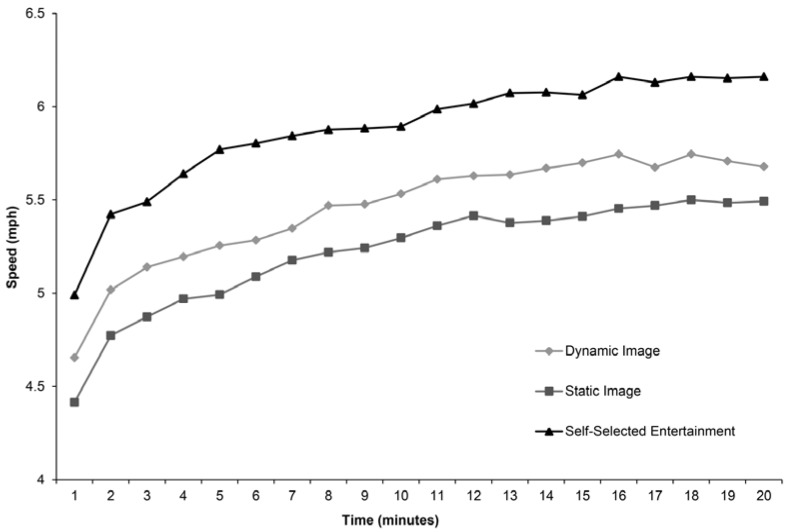
The mean minute-by-minute running speed in the three different conditions.

The mean minute-by-minute running speed in the three conditions is presented in Figure 2. The three exercise groups presented different running speeds, however with similar tendencies, i.e., gradually increasing speed throughout the twenty-minute period.

### 3.2. Emotional Variables

Descriptive data of pre-and-post run scores of five subscales of SEQ of three different conditions are displayed in Table 2. The scores of each subscale range from 0 to 4. 

#### 3.2.1. Happiness

**Figure 3:**
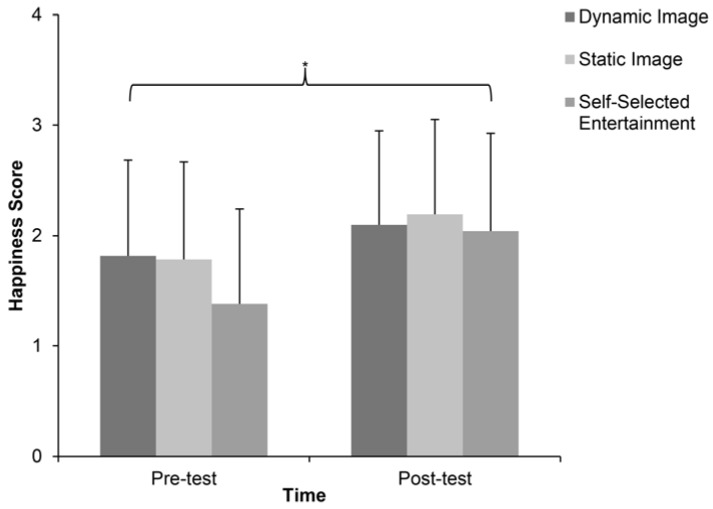
Pre-and-post scores on happiness scale in three conditions (mean ± SD). * indicating the time effect, *p* < 0.05.

Time had a main effect on reported feelings of happiness (Figure 3.). People felt happier after running (pre-scores 1.67 ± 0.88; post-scores 2.11 ± 0.86; *F* (1, 29) = 27.185, *p* < 0.05, *ƞp*^2^ = 0.484). There was also a main effect for exercise design on reported feelings of happiness, *F* (2, 58) = 3.656, *p* < 0.05, *ƞp*^2^ = 0.112 when the data of pre-and-post in each condition were pooled. The post hoc analysis indicated that participants felt happier in the dynamic image condition (1.958 ± 0.114), *p* < 0.05 and in the static image condition (1.987 ± 0.147), *p* < 0.05, than in the self-selected entertainment condition (1.713 ± 0.142; Figure 3). There were no interaction effects between time and exercise design on reported feelings of happiness, *F* (2, 58) = 2.337, *p* > 0.05, *ƞp*^2^ = 0.075.

#### 3.2.2. Anxiety

Time had a main effect on reported feelings of anxiety, *F* (1, 29) = 16.256, *p* < 0.05, *ƞp*^2^ = 0.259 with participants feeling less anxious after running (pre 0.471 ± 0.066; post 0.218 ± 0.039) regardless of PA designs. There was no main effect of designs on reported feelings of anxiety, *F* (2, 58) = 0.190, *p* > 0.05, *ƞp*^2^ = 0.047, which showed that participants reported a similar level of anxiety across three designs. There were no interaction effects between time and exercise design on reported feelings of anxiety, *F* (2, 58) = 0.322, *p* > 0.05, *ƞp*^2^ = 0.016.

#### 3.2.3. Dejection

Time had a main effect on reported feelings of dejection, *F* (1, 29) = 10.296, *p* < 0.05, *ƞp*^2^ = 0.262. Participants felt less dejected after running (pre 0.162 ± 0.035; post 0.047 ± 0.014) regardless of designs. There was no main effect for condition on reported feelings of dejection, which indicated that participants reported a similar level of dejection across three designs *(F* (2, 58) = 0.645, *p* > 0.05, *ƞp*^2^ = 0.022). There was also no interaction between time and exercise designs on reported feelings of dejection, *F* (2, 58) = 0.356, *p* > 0.05, *ƞp*^2^ = 0.012.

#### 3.2.4. Anger

Time had a main effect on reported feelings of anger, *F* (1, 29) = 4.563, *p* < 0.05, *ƞp*^2^ = 0.136 with participants feeling less angry after running (pre 0.161 ± 0.049; post 0.069 ± 0.024) regardless of design. There was no main effect for condition on reported feelings of anger, *F* (2, 58) = 0.190, *p* > 0.05, *ƞp*^2^ = 0.047, which indicated that people report a similar level of anger across three designs. There were no interactions between time and exercise design on reported feelings of anger, *F* (2, 58) = 0.322, *p* > 0.05, *ƞp*^2^ = 0.011.

#### 3.2.5. Excitement

Time had a main effect on reported feelings of excitement, *F* (1, 29) = 97.054, *p* < 0.05, *ƞp*^2^ = 0.770. Participants felt more excited after running (pre 0.947 ± 0.092; post 1.906 ± 0.136) regardless of the condition. There was no main effect for condition on reported feelings of excitement, which indicated people reported similar levels of excitement across all designs (*F* (2, 58) = 0.459, *p* > 0.05, *ƞp*^2^ = 0.016). There were no interactions between time and exercise condition on reported feelings of excitement, *F* (2, 58) = 1.318, *p* > 0.05, *ƞp*^2^ = 0.043. 

## 4. Discussion

The aim of this paper was to examine physical and emotional effects of the design of different exercise environments, using preferred entertainment and the presence of nature (using a static and dynamic image), without imposing the same intensity levels of physical activity (PA) on all participants. For physical outcomes, the self-selected entertainment condition resulted in greater physical benefits. That is, participants ran longer distances with a higher heart rate (HR) value, compared to the nature-based exercise designs. Previous studies investigating the physical benefits of indoor exercise in the presence of nature have shown inconsistent findings, with some studies advocating enhanced physical effects, such as lowing perceived exertion [14,15] and blood pressure [12,13]. In contrast, research has shown no differences in energy expenditure [6,26], perceived exertion [26] and HR [12,26]. The varying benefits of green PA found in previous studies may be linked to the control conditions to which green PA was examined against. In the present study, by introducing a more ecological representative control conditions (i.e., self-selected entertainment) rather than imposing a less representative control condition, like asking participants to view a blank wall, we were able to examine the effects of introducing a nature-based environment compared a typical gym environment. As participants of gym-based PA would typically engage in the exercise experience using self-selected entertainment, rather than viewing urban images or a blank wall, our results suggested that, over longer running distances using self-selected entertainment could be beneficial if an individual’s main goal when exercising is to enhance physical performance. 

Although the findings revealed that the use of self-selected entertainment resulted in participants running farther than in the two nature designs, with a higher HR, it is worth noting that greater happiness was reported in the two nature-based exercise designs compared to the self-selected entertainment PA. All participants accrued emotional benefits with decreases in anger, dejection and anxiety and increased excitement after the run in all PA designs using indoor treadmill running. These findings suggested that nature-based exercise designs are just as effective as preferred exercise conditions with which participants were most familiar. The two nature-based designs showed stronger effects on happiness compared to self-selected entertainment conditions. The enhanced happiness scores observed in the nature-based PA designs indicate that using nature images for exercise is of some value, since if participants experience greater happiness after exercising they would be more likely to prolong exercise duration or benefit from exercise adherence [34]. A positive exercise experience is more likely to be associated with maintenance of future physical activity participation [35], which can also help in promoting physical activity.

Inconsistent results in the literature might also be because of the use of different modes of PA (e.g., cycling and running), different exercise durations (e.g., 5 min, 15 min and 20 min) and different intensity levels (e.g., maintain 70–80 rpm or cycling at 50% personal peak power output). While the majority of previous studies controlled exercise intensity, based on each runner’s maximum energy output, we intentionally did not regulate the intensity of PA. Instead we designed a study which would allow us to find out how people interacted with different environmental designs by detecting information from a specific environment. An important consideration when interpreting the finding that the self-selected entertainment condition increased running distance compared to nature designs relates to the type of nature conditions presented to participants. In the static nature image condition, participants detected the same visual information with minor changes from the physical environment over twenty minutes. In this case, the same visual information from the static image might have become less functional, without providing further inspiration or encouragement for physical activity. This interpretation supports results of previous research which examined the physiological benefits of nature exposure during exercise and found the first 5 min was more efficient than the second 5 min in eliciting improvements in the recovery process following a stressor [39]. Participants might detect richer visual information from the dynamic images during running. However, the suitability of the information offered by the dynamic images might also need to be considered in further work on treadmill running [6]. The information perceived from the video might not have closely matched the physical task, i.e., treadmill running, as the recording was made while cycling in the park. This might have been a distraction for physical performance on a treadmill. Participants might have found that the richer information in the dynamic image condition lead to some dissonance between perception and action. In the self-selected entertainment condition, people chose acoustic or visual-acoustic information which constantly offered rich information acoustically and visually during the run. Further, the majority of self-selected entertainment chosen in this study was listening to music. Previous research has investigated the effects of different types of music (e.g., self-selected, motivational and simulative), demonstrating benefits, such as encouraging physical performances, enhancing enjoyment, reducing ratings of perceived exertion and improving energy efficiency [7,19,40,41,42]. The findings in our study, regarding better physical outcomes when running with self-selected entertainment, are aligned with previous research. 

Greater perceived happiness was found when people exercised in the nature-based designs compared to the self-preferred, familiar entertainment condition. This finding might be because these two nature-based exercise designs encouraged participants to engage more with the presented information, rather than focusing on physical performance and running. The exercise experience under the nature conditions might have been more dissociative, while running with music might be more associative in focusing on exercise intensity during PA [43]. Further investigations, involving interviewing participants post exercise, might be able to shed further light on this assumption. All participants experienced less anxiety, less dejection, more excitement and less anger in all three exercise designs after twenty minutes of running supports the notion that exercise has positive emotional benefits. Based on the results, the acoustic or visual-acoustic information in the self-selected entertainment condition aided runners’ physical performance outcomes while the visual nature-based information would be more beneficial to emotional wellbeing. With regards to the design of typical gym exercise conditions, there are different types of self-selected entertainment used in this study which might lead to different exercise outcomes. Future studies could consider focusing on entertainment choices in a highly specific way, without reducing the representative design of the research. Further studies could explore presentation of images from different types of nature spaces, such as beaches, oceans, and forest trails as exercise environments and different sources of information, e.g., nature sounds, could be influential and need to be examined. 

## 5. Conclusions

In conclusion, this study advances our understanding of the physical and emotional effects of different affordances in exercise designs for indoor treadmill running. However, there is much that still needs to be explored, such as different types of media or different contents of media, might accrue different effects among different age groups. Different methods, such as qualitative interviews, can also be used in future research to explore the data from a different perspective, such as the participants’ perspectives on engagement with the physical activity (PA) environment under different designs.

## Figures and Tables

**Table 1:** The mean ± SD running distances and Heart Rate (HR) values in the three different conditions.

Variables	*N*	Dynamic Image	Static Image	Self-Selected Entertainment
Distance (m)	30	2891.6 ± 631.4	2767.2 ± 662.6 *	3066.8 ± 688.5 *
HR (bpm)	24	141 ± 18 *	138 ± 21 *	147 ± 188 *

*: indicated that *p* < 0.05.

**Table 2:** The pre-and -post run scores of five subscales of Sport Emotion Questionnaires (SEQ) of three different conditions (mean ± SD).

Variables	*N*	Dynamic Image	Static Image	Self-Selected Entertainment
Anxiety Pre-test	30	0.48 ± 0.68	0.52 ± 0.74	0.40 ± 0.52
Anxiety Post-test	30	0.17 ± 0.26	0.22 ± 0.34	0.26 ± 0.31
Dejection Pre-test	30	0.20 ± 0.45	0.11 ± 0.15	0.17 ± 0.33
Dejection Post-test	30	0.04 ± 0.11	0.02 ± 0.08	0.06 ± 0.16
Excitement Pre-test	30	1.09 ± 1.01	0.95 ± 1.07	0.80 ± 1.03
Excitement Post-test	30	1.76 ± 0.88	2.05 ± 0.93	1.89 ± 0.72
Anger Pre-test	30	0.20 ± 0.57	0.15 ± 0.46	0.12 ± 0.26
Anger Post-test	30	0.06 ± 0.18	0.07 ± 0.20	0.06 ± 0.18
Happiness Pre-test	30	1.81 ± 0.86	1.78 ± 0.88	1.38 ± 0.85
Happiness Post-test	30	2.10 ± 0.84	2.19 ± 0.86	2.04 ± 0.88
